# Order-disorder transition in active nematic: A lattice model study

**DOI:** 10.1038/s41598-017-07301-w

**Published:** 2017-08-01

**Authors:** Rakesh Das, Manoranjan Kumar, Shradha Mishra

**Affiliations:** 1 S N Bose National Centre for Basic Sciences, Block JD, Sector III, Salt Lake, Kolkata, 700106 India; 2 Department of Physics, Indian Institute of Technology (BHU), Varanasi, 221005 India

## Abstract

We introduce a lattice model for active nematic composed of self-propelled apolar particles, study its different ordering states in the density-temperature parameter space, and compare with the corresponding equilibrium model. The active particles interact with their neighbours within the framework of the Lebwohl-Lasher model, and move anisotropically along their orientation to an unoccupied nearest neighbour lattice site. An interplay of the activity, thermal fluctuations and density gives rise distinct states in the system. For a fixed temperature, the active nematic shows a disordered isotropic state, a locally ordered inhomogeneous mixed state, and bistability between the inhomogeneous mixed and a homogeneous globally ordered state in different density regime. In the low temperature regime, the isotropic to the inhomogeneous mixed state transition occurs with a jump in the order parameter at a density less than the corresponding equilibrium disorder-order transition density. Our analytical calculations justify the shift in the transition density and the jump in the order parameter. We construct the phase diagram of the active nematic in the density-temperature plane.

## Introduction

Self-propelled particles compose an interesting type of the active systems^1–5^ where each particle extracts energy from its surroundings and dissipates it through motion and collision. Their examples range from very small intracellular scale to larger scales^6–20^. Also many artificially designed systems, e.g., vibrated granular media^21–24^, active polar disks^25^, active colloids^26–29^ imitate the physics of the active systems. If $\hat{{\rm{n}}}$ is the average alignment direction of a collection of such active particles, and the system remains invariant under the transformation $\hat{{\rm{n}}}\to -\hat{{\rm{n}}}$, it is called ‘active nematic’. Activity introduces many interesting properties which are absent in their thermal equilibrium counterparts. One of such interesting features is the presence of large density fluctuation in the ordered active nematic^30^. Density is a key control parameter in various experiments and numerical simulations. Earlier studies on equilibrium nematic for a fixed temperature show the isotropic to nematic transition at some critical density^31^. However, the effect of the density fluctuation in the active nematic is not well understood.

Most of the previous studies of the active systems are done either by using the coarse-grained hydrodynamic equations of motion^32^ or microscopic rule based numerical simulation of agent based point particles^33^ or Brownian dynamic simulation^34^. Here we introduce a lattice model for a two-dimensional active nematic, explore various states of the system in the density-temperature plane, and compare it with the corresponding equilibrium model. In general, lattice model itself is interesting for development of simplified theories, and provides insight into complex systems. Our model is analogous to the previous lattice model of polar active spins^35, 36^; but we include volume exclusion to avoid multiple occupancy on single site. Such volume exclusion limits the motion of particles towards an occupied neighbouring site, and introduces new features, e.g., typical pattern formation^20, 37^, density induced motility^38^ in the system.

**Figure 1:**
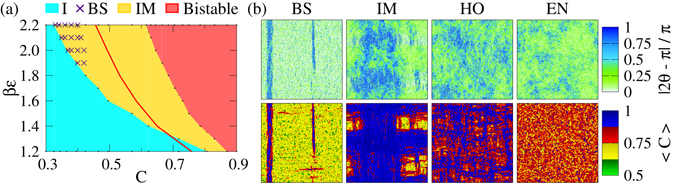
Phase diagram. (**a**) Phase diagram for both the equilibrium and the active nematic in the density - inverse temperature plane. The equilibrium system remains in the isotropic (EI) state in the low density regime (on the left of the solid line) and in the nematic (EN) state in the high density regime (on the right of the solid line). The active nematic goes from the disordered isotropic (I) state to the locally ordered inhomogeneous mixed (IM) state with increasing density or decreasing temperature. The I - IM transition occurs with the appearance of clear bands (BS) in the low temperature regime. In the high density regime the active nematic shows bistability between the IM and the homogeneous globally ordered (HO) state. (**b**) Upper panel shows particle inclination towards the horizontal direction. Colour bar ranging from zero to one indicates vertical to horizontal orientation, respectively. BS is the banded state configuration shown for (*βε*, *C*) = (2.0, 0.38). IM, HO and EN state configurations are shown for (*βε*, *C*) = (2.0, 0.78). Lower panel shows the coarse-grained density in the respective states.

**Figure 2:**
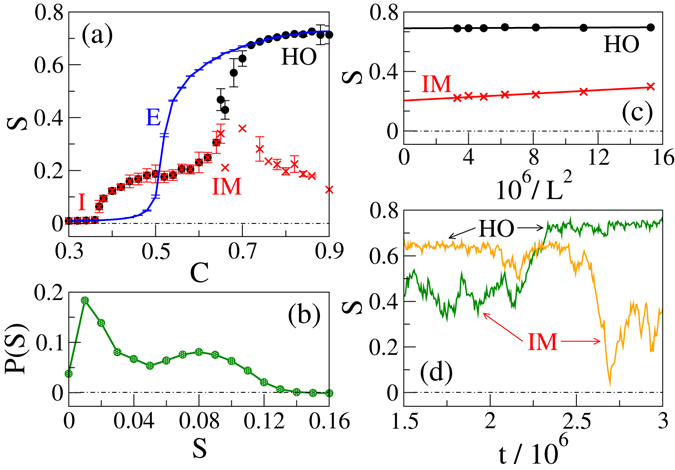
Disorder - order transition. (**a**) Scalar order parameter versus packing density plot for 400 × 400 system size at *βε* = 2.0. The equilibrium system (E, solid line) shows continuous isotropic to nematic state transition with increasing density. The active system goes from the isotropic (I) state to the locally ordered inhomogeneous mixed (IM, ×) state. In the high density regime, the system shows bistability between the IM state and the homogeneous globally ordered (HO, •) state. (**b**) The I to IM transition at low temperature occurs with a jump in *S* where the particles form bands (BS). Distribution of the scalar order parameter near the I - BS transition at (*βε*, *C*) = (2.0, 0.37) shows two peaks. (**c**) Finite size scaling of *S* for both the HO and the IM state at (*βε*, *C*) = (2.0, 0.76). (**d**) Order parameter time series show that the active system flips in between the HO and the IM state in the bistable regime. Two time series are shown for two different parameter values in the high density regime.

We construct a phase diagram for the active nematic in the density-temperature plane, as shown in Fig. 1(a). There we observe - (i) disordered isotropic (I) state in low density regime, (ii) locally ordered inhomogeneous mixed (IM) state in intermediate density regime, and (iii) bistability between the IM and a homogeneous globally ordered (HO) state in high density regime. In contrast to the continuous isotropic to nematic (I-N) transition in the equilibrium system, the I to IM state transition in the active nematic in the low temperature regime occurs with a jump in order parameter, as shown in Fig. 2(a). This transition occurs at a density lower than the equilibrium critical value, and the system forms clear bands (BS) in this regime. We finally justify the jump in the order parameter and the shift in the transition density by analytical study of the coarse-grained hydrodynamic equations written for the active model.

## Model

**Figure 3:**
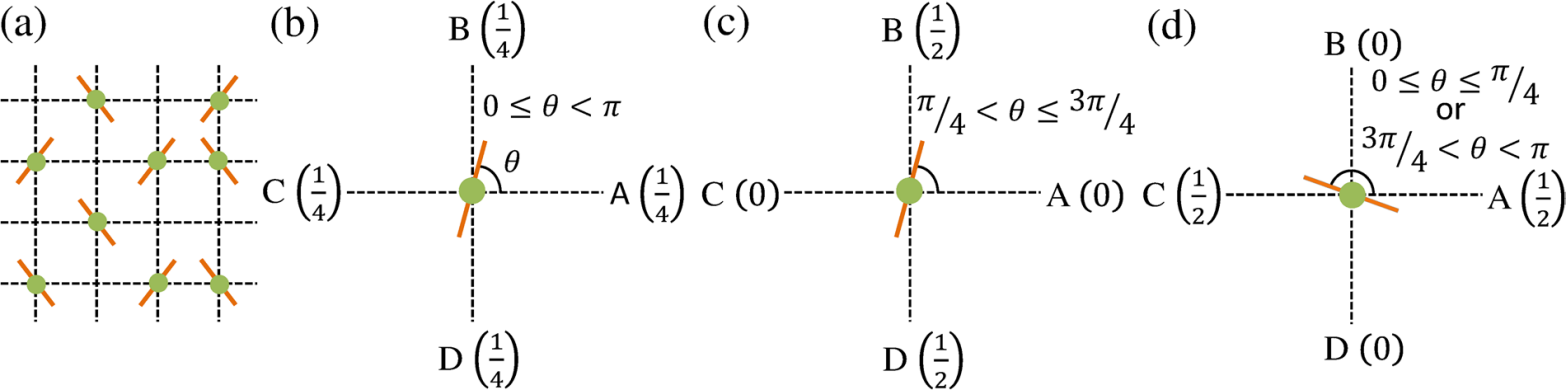
Model figure. (**a**) Two dimensional square lattice with occupied (*n* = 1) or unoccupied (*n* = 0) sites. Filled circles indicate the occupied sites. Inclinations of the rods towards the horizontal direction show respective particle orientations *θ* ∈ [0,*π*]. (**b**) Equilibrium move: particle can move to any of the four neighbouring sites with equal probability 1/4. (**c**,**d**) Active move: particle can move to either of its two neighbouring sites with probability 1/2, if unoccupied, in the direction it is more inclined to, i.e., along *BD* in (**c**), and *AC* in (**d**).

We consider a collection of apolar particles on a two dimensional square lattice, as shown in schematic diagram Fig. 3(a). Occupation number ‘*n*_*i*_’ of the *i* ^*th*^ lattice site can take values 1 (occupied) or 0 (unoccupied). Orientation *θ*_*i*_ of apolar particle at the *i* ^*th*^ site can take any value between 0 and *π*. The model follows two sequential processes at every step; first, a particle moves to a nearest neighbouring site with *some probability*, and then orientation of the particle is updated based on its nematic interaction with its nearest neighbours. We define two kinds of models on the basis of particle movement: (i) ‘Equilibrium model’ (EM) - particle moves with equal probability 1/4 to any of the four neighbouring sites (Fig. 3(b)), (ii) ‘Active model’ (AM) - in this model particle movement occurs in two steps. First, it chooses a direction along which it is more inclined. As shown in Fig. 3(c,d), it chooses the direction of movement along *BD* if $\pi /4 < \theta \le 3\pi /4$ and along *AC* otherwise. In the second step, it moves to a randomly selected site between the two nearest neighbouring sites along the chosen direction. For example, if *BD* is selected as the direction of movement, then the particle moves to randomly selected site *B* or *D* in the second step. In both the models, we consider volume exclusion, i.e., particle movement is allowed only if the selected site is unoccupied.

In both the models, the particles also interact with their nearest neighbours. The interaction depends on the relative orientation of the particles and is represented by a modified Lebwohl-Lasher Hamiltonian^39^

1 $$ {\mathcal H} =-\varepsilon \sum _{ < ij > }{n}_{i}{n}_{j}\,\cos \,\mathrm{2(}{\theta }_{i}-{\theta }_{j})$$

where *ε* is the interaction strength between two neighbouring particles. The interaction in equation (1) governs the orientation update of the particle. We employ Metropolis Monte-Carlo (MC) algorithm^40^ for orientation update of the particle after the movement trial. In both the models, an order parameter defining the global alignment of the system does not remain conserved during the MC orientation update described above. In actual granular or biological systems where mutual alignment emerges because of steric repulsion, orientation of particles need not to follow a conservation law. An order parameter defined by coarse-graining the orientation in our present model is a class of non-conserved order parameter: *Model A* as described by Hohenberg and Halperin^41^.

Both the models EM and AM comprise of two different physical aspects - motion of the particles and nematic interaction amongst the nearest neighbours. If the particles are not allowed to move, the models reduce to an apolar analogue of the diluted XY-model with nonmagnetic impurities^42^, where impurities and spins are analogous to vacancies and particles, respectively. However, unlike the diluted XY-model, particles in these models are dynamic. In the EM, the particle diffuses to neighbouring sites, whereas it moves anisotropically in the AM. The anisotropic movement of the active particles arises in general because of the self-propelled nature of the particles in many biological^43^ and granular systems^21, 22^. This move produces an active curvature coupling current in the coarse-grained hydrodynamic equations of motion^30, 32^. The AM does not satisfy the detailed balance principle^40^, because of the orientation update after the anisotropic movement. The coupling of the particle movement with the orientation update in our active model is analogous to the active Ising spin model introduced by Solon and Tailleur^35, 36^, where the probabilistic flip of the spins is an equilibrium process, whereas the out-of-equilibrium aspect of the model is attributed to the anisotropic movement probability of the spins. However, their orientation update algorithm^35, 36^ is similar to kinetic Monte-Carlo, whereas we use Metropolis Monte-Carlo algorithm to update particle orientation.

### Numerical Study

We consider a collection of *N* particles with random orientation *θ* ∈ [0, *π*] homogeneously distributed on a *L* × *L* lattice (*L* = 256,400,512) with periodic boundary. Packing density of the system is defined as *C* = *N*/(*L* × *L*). We choose a particle randomly, move it to a neighbouring site obeying exclusion, and then update its orientation using Metropolis algorithm. In each iteration, we repeat the same process for *N* number of times, and we use 1.5 × 10^6^ iterations to achieve the steady state of the system. We obtain the steady state results by averaging the observables over next 1.5 × 10^6^ iterations and use more than twenty realisations for better statistics.

The ordering in the system is characterised by a scalar order parameter defined as

2 $$S=\sqrt{{(\frac{1}{N}\sum _{i}{n}_{i}\cos \mathrm{(2}{\theta }_{i}))}^{2}+{(\frac{1}{N}\sum _{i}{n}_{i}\sin \mathrm{(2}{\theta }_{i}))}^{2}}.$$

It is proportional to the positive eigenvalue of the nematic order parameter Q^31^. It takes the minimum value 0 in the disordered state and the maximum value 1 in the complete ordered state. First we study the EM as a function of inverse temperature *β* = 1/*k*_*B*_ *T* for different packing densities. As shown in Supplementary Figure S1, the system shows disordered isotropic to nematic state (I-N) transition with decreasing temperature. In contrast to the first order I-N transition in the equilibrium Lebwohl-Lasher model in three dimensions^39, 44^, we find continuous transition for the EM defined in two dimensions. The observed nature of transition supports the study by Mondal and Roy^45^. Similar to the diluted XY-model^42^, the critical inverse temperature *β*_*c*_(*C*) increases with density in the EM.

### Phase diagram

We construct phase diagram for both the equilibrium model and the active model on the density-temperature plane. As shown in Fig. 1(a), two distinct states appear in the EM - (i) an equilibrium isotropic (EI) state on the left side of the red boundary and (ii) an equilibrium nematic (EN) state on the right side of the red boundary. In the EI state, particles remain disordered and homogeneously distributed throughout the system. Consequently, the scalar order parameter $S\simeq 0$ in this state. With increasing density or decreasing temperature the particles get mutually ordered and form the EN state (*S* > 0). As shown in Fig. 2(a), for a fixed temperature the scalar order parameter increases continuously with increasing density, and the system enters into the nematic state. Both the particle orientation and the coarse-grained density remain homogeneous in the EN state, as shown in the real space snapshot Fig. 1(b).

Similar to the EM, the active system remains in a homogeneous disordered isotropic (I) state in the high temperature and/or low packing density regime (cyan coloured regime in the phase diagram Fig. 1(a)). With increasing density or decreasing temperature, beyond the I state, the active system enters into an inhomogeneous mixed (IM) state (golden regime in the phase diagram Fig. 1(a)), where locally ordered high-density domains coexist with disordered low-density regions. In the low temperature regime (*βε* ∈ [1.9, 2.2]), the I to IM state transition with increasing *C* occurs with a jump in the scalar order parameter *S*, as shown in Fig. 2(a). In the very beginning of the IM state, as indicated by cross symbols in Fig. 1(a), we find a banded state (BS) in the low temperature regime, where particles cluster and align themselves within a strip to form band. However, out of the strip the system remains disordered with low local density, as shown in the real space snapshot Fig. 1(b). On further increment of the packing density *C*, bands formed in different directions start mixing leaving the system with many locally ordered high density patches separated by low density disordered regions. Typical real space snapshots for the orientation and the coarse grained density in the IM state are shown in Fig. 1(b). The jump in the *S*-*C* curve reduces with increasing temperature, and no bands appear in the high temperature (βε < 1.9) regime.

**Figure 4:**
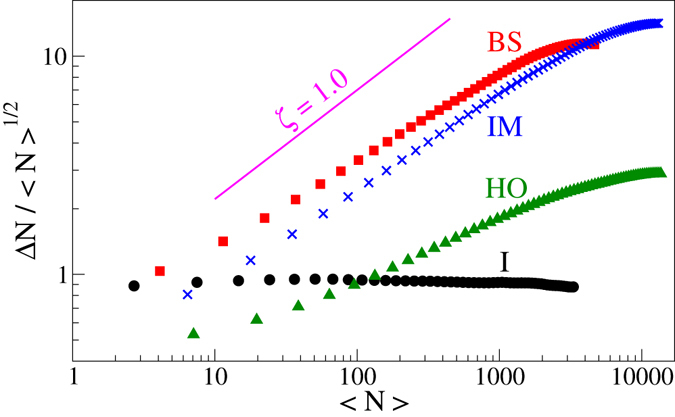
Density fluctuation ${\rm{\Delta }}N=\sqrt{ < {N}^{2} > - < N{ > }^{2}}$. All the active ordered states show large density fluctuation obeying the relation ${\rm{\Delta }}N\sim  < N\,{ > }^{\zeta }$ with *ζ* > 1/2. The active disordered isotropic state shows normal density fluctuation with *ζ* = 1/2.

Figure 2(a) shows that the I to BS transition occurs in the low temperature regime with a jump in *S* at a density lower than the corresponding equilibrium I-N transition density *C*_*IN*_. These bands appear because of the large activity strength. A linear stability analysis, as detailed later in this paper, shows that the large activity strength induces an instability in the disordered isotropic state. This instability goes away for small activity strength or at high temperature. We also do a renormalised mean field calculation of an effective free energy written for the active nematic. The calculation predicts a jump in the scalar order parameter and shows a shift in the disordered (*S* = 0) to ordered (*S* ≠ 0) state transition density. Both the jump in *S* and the shift in the transition density reduce with the activity strength or increasing temperature. The I to BS transition is a first order transition. The shift in the disorder-order transition point is a common feature of the active systems. For large activity and low temperature, if the system density is above a certain value but less than *C*_*IN*_, the large density fluctuation present in these systems causes local alignment with local density higher than *C*_*IN*_. Large density fluctuation is an intrinsic feature of the active systems, and as shown in Fig. 4, we also observe the same in the ordered active states in our model. Due to activity, these locally ordered regions move anisotropically and combine with nearby region with similar local ordering. So larger ordered region forms at mean density lower than the equilibrium I-N transition density. Therefore, we find a disordered to ordered state transition at a lower density. For large activity strength, I-BS transition occurs with the jump in scalar order parameter. In our numerical study, we calculate the probability *P*(*S*) of the scalar order parameter averaging over many iterations and realisations near the I-BS transition point. Figure 2(b) shows *P*(*S*) has two peaks, which further supports the first order I-BS transition for large activity strength.

In the high density regime (red coloured regime in the phase diagram Fig. 1(a)), the AM shows bistability, i.e., it can be either in the locally ordered IM state or in a homogeneous globally ordered (HO) state. As shown in Fig. 2(a), the *S*-*C* curve for fixed temperature bifurcates in the high density regime; the lower branch corresponds to the earlier discussed IM state, whereas the higher branch indicates the existence of the globally ordered state. Figure 1(b) shows that the system possesses less density inhomogeneity in the HO state compared to the IM state. A finite size scaling of both the HO and the IM state, as shown in Fig. 2(c), shows that the active nematic possesses non-zero finite order in both these states. Order parameter time series shown in Fig. 2(d) confirms the bistability of the system in the high density regime. Bistability is not generally seen in other agent based numerical simulations of point particles^33^; it appears because of finite filling constraint of the model. This feature can be suppressed if we allow more than one particle to sit together. In the complete filling limit *C* = 1.0, the AM is equivalent to the EM, and it shows the globally ordered HO state only.

### Two-point orientation correlation

**Figure 5:**
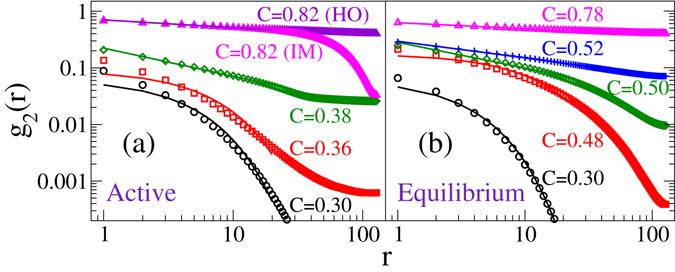
Two-point orientation correlation shown for *βε* = 2.0 on log-log scale. (**a**) Active model: *g*_2_(*r*) decays exponentially at low density (○, ◽) and algebraically at high density (◊, Δ). In the bistable regime at high density (Δ), *g*_2_(*r*) decays algebraically in the HO state and abruptly in the IM state. (**b**) Equilibrium model: *g*_2_(*r*) decays exponentially at low density (○, ◽) and algebraically at high density (◊, +, Δ). Continuous lines are the respective fits, fitted for more than one decade.

We further characterise various states on the basis of the two-point orientation correlation in the different states of the equilibrium and the active nematic. It is defined as ${g}_{2}(r)= < {\sum }_{i}{n}_{i}{n}_{i+r}$ $\cos [2({\theta }_{i}-{\theta }_{i+r})]/{\sum }_{i}\,{n}_{i}^{2} >$ where *r* represents interparticle distance, and *<*⋅*>* signifies an average over many realisations. Figure 5(a,b) show *g*_2_(*r*) versus *r* plots on log-log scale for the AM and the EM, respectively, for a fixed inverse temperature *βε* = 2.0. In the AM, *g*_2_(*r*) decays exponentially at low packing density *C* < 0.38, i.e., in the isotropic state. Therefore, the active isotropic is a short-range-ordered (SRO) state. In the BS at *C* = 0.38, *g*_2_(*r*) decays following a power law. Therefore, the system is in a quasi-long-range-ordered (QLRO) state. Ordering increases with density. At high packing density, correlation function confirms the bistability in the active system. At *C* = 0.82, *g*_2_(*r*) shows power law decay in the HO state, whereas in the IM state *g*_2_(*r*) decays abruptly after a distance *r*. The abrupt change in *g*_2_(*r*) at a certain distance indicates the presence of locally ordered clusters in the IM state. In contrast, the equilibrium system shows a transition from SRO (exponential decay) isotropic state at low density *C* $\lesssim$ 0.48 to QLRO (power law decay) nematic state at high density *C* $\gtrsim$ 0.50.

### Orientation distribution and autocorrelation of the mean orientation

**Figure 6:**
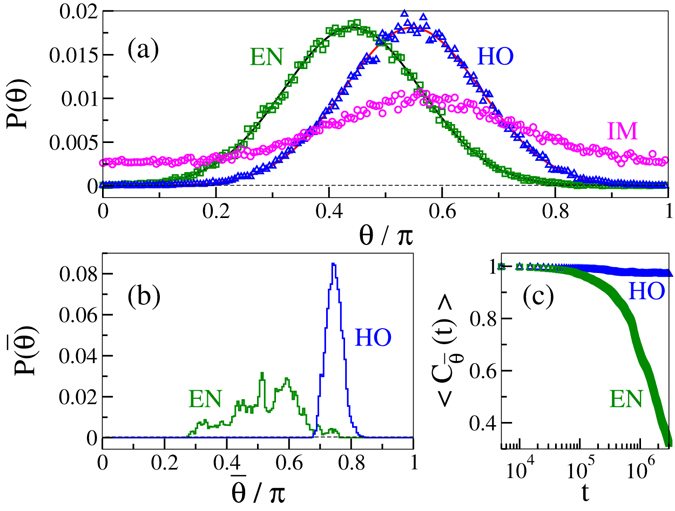
Steady state characteristics of high density states. (**a**) Orientation distribution *P*(*θ*) of particles calculated from one snapshot in the steady state. *P*(*θ*) fits with Gaussian distribution (continuous lines) for both the HO and the EN states. The IM state shows broad distribution of *θ*. (**b**) Distribution of the mean orientation *P*($\bar{\theta }$) calculated from $\bar{\theta }$ of each snapshot in the steady state. *P*($\bar{\theta }$) is broad for the EN state in comparison to the HO state. (**c**) Steady state autocorrelation *C* ${}_{\bar{\theta }}$(*t*) of the mean orientation of the system. All plots are shown for (*βε*, *C*) = (2.0, 0.80).

We compare the steady state properties of the active and the equilibrium models in the high density limit. First we calculate the steady state (static) orientation distribution *P*(*θ*) from a snapshot of particle orientation *θ*. As shown in Fig. 6(a), both the active HO and the equilibrium nematic show Gaussian distribution of orientation. Peak position of *P*(*θ*) for both the EN and the HO state can appear at any point between 0 and *π* because of the continuous broken rotational symmetry of the Hamiltonian shown in equation (1). Data shown in Fig. 6(a) is for one realisation only, and for other realisations also the distribution *P*(*θ*) remains Gaussian with peak at other *θ* values. Therefore, orientation fluctuation of the particles in the active HO state is same as in the equilibrium nematic state. The distribution *P*(*θ*) in the IM state is very broad and spans over the whole range of orientation. Therefore, the system possess no global ordering in the IM state.

We also calculate the time averaged distribution *P*($\bar{\theta }$) of mean orientation of all the particles in the active HO and the equilibrium nematic states. The mean orientation $\bar{\theta }$(*t*) of all particles is calculated for each iteration time *t* in the steady state. The distribution *P*($\bar{\theta }$) of the mean orientation is obtained from these $\bar{\theta }$(*t*) data. This distribution is a measure of the fluctuation in the global orientation of the particles in the steady state. As shown in Fig. 6(b), *P*($\bar{\theta }$) in the active HO state is narrow in comparison to the broad distribution in the EN state. We also calculate the autocorrelation of the mean orientation ${{\rm{C}}}_{\bar{\theta }}(t)= < \frac{1}{t}{\sum }_{\tau =1}^{t}cos[2\{\bar{\theta }({t}_{0})-\bar{\theta }({t}_{0}+\tau )\}] >$ in the steady state. As shown in Fig. 6(c), ${{\rm{C}}}_{\bar{\theta }}(t)$ decreases with time in the EN state, but remains unchanged in the active HO state. Both these results imply that the fluctuation in the global orientation direction $\bar{\theta }$ in the active HO state is small compared to the EN state. We do not calculate the mean orientation $\bar{\theta }$ in the active IM state, because the system possesses no global ordering in this state.

### Phenomenological approach to understand low density states of the active model

In this section we write the hydrodynamic equations of motion for the active model and characterise the low density states of the system. The equations of motion for the slow variables of the system, i.e., the number density $\wp ({\bf{r}},t)={\sum }_{l}\delta ({\bf{r}}-{{\bf{R}}}_{l}(t))$ and the order parameter ${{\rm{\Pi }}}_{ij}({\bf{r}},t)={\rm{\wp }}({\bf{r}},t){\boldsymbol{Q}}{}_{ij}({\bf{r}},t)={\sum }_{l}({{\bf{m}}}_{li}{{\bf{m}}}_{lj}-\frac{1}{2}{\delta }_{ij})\delta ({\bf{r}}-{{\bf{R}}}_{l}(t))$ are as follows^30, 32^:

3 $${\partial }_{t}\wp ={a}_{0}{\nabla }_{i}{\nabla }_{j}{{\rm{\Pi }}}_{ij}+{D}_{\wp }{\nabla }^{2}\wp $$

and

4 $${{\rm{\partial }}}_{t}{{\rm{\Pi }}}_{ij}=\{{\alpha }_{1}({\rm{\wp }})-{\alpha }_{2}({\rm{\Pi }}:{\rm{\Pi }})\}{{\rm{\Pi }}}_{ij}+\beta ({{\rm{\nabla }}}_{i}{{\rm{\nabla }}}_{j}-\frac{1}{2}{\delta }_{ij}{{\rm{\nabla }}}^{2}){\rm{\wp }}+{D}_{{\rm{\Pi }}}{{\rm{\nabla }}}^{2}{{\rm{\Pi }}}_{ij}.$$

Here **R**_*l*_(*t*) represents position of the particle *l*, and **m**_*l*_ = (cos*θ*_*l*_, sin*θ*_*l*_) is the unit vector along the orientation *θ*_*l*_. The total number of particles being a conserved quantity of the system, equation (3) represents a continuity equation ${\partial }_{t}\wp =-\nabla \cdot J$ where the current ${J}_{i}=-{a}_{0}{\nabla }_{j}{{\rm{\Pi }}}_{ij}-{D}_{\wp }{\nabla }_{i}\wp$. The first term of *J*_*i*_ consists of two parts: an anisotropic diffusion current ${{\bf{J}}}_{p1}\propto \,\,{\bf{Q}}{}_{ij}{\nabla }_{i}\wp$ and an active curvature coupling current ${{\bf{J}}}_{a}\propto {a}_{0}\wp {\nabla }_{j}{\bf{Q}}{}_{ij}$ where *a*_0_ is the activity strength of the system. The second term represents an isotropic diffusion ${{\bf{J}}}_{p2}\propto \nabla \wp$. The *α* terms in equation (4) represent mean field alignment in the system. We choose ${\alpha }_{1}(\wp )=(\frac{\wp }{{\wp }_{IN}}-1)$ as a function of density that changes sign at some critical density ${\wp }_{IN}$. The *β* term represents coupling with density. The last term represents diffusion in order parameter that is written under equal elastic constant approximation for two-dimensional nematic. The steady state solution $\wp ({\bf{r}},t)={\wp }_{0}$ and Π(**r**, *t*) = Π_0_, where ${{\rm{\Pi }}}_{0}=\sqrt{\frac{{\alpha }_{1}({\wp }_{0})}{{\alpha }_{2}}}$, of equations (3) and (4) represents a homogeneous ordered state for ${\alpha }_{1}({\wp }_{0}\mathrm{) > 0}$ at ${\wp }_{0} > {\wp }_{IN}$, and a disordered isotropic state for ${\alpha }_{1}({\wp }_{0}\mathrm{) < 0}$ at ${\wp }_{0} < {\wp }_{IN}$.

We study the linear stability of the disordered isotropic state (Π_0_ = 0) by examining the dynamics of spatially inhomogeneous fluctuations $\delta \wp ({\bf{r}},t)=\wp ({\bf{r}},t)-{\wp }_{0}$, *δ*Π_11_ = Π_11_(**r**, *t*), and *δ*Π_12_ = Π_12_(**r**, *t*). We obtain the linearised coupled equations of motion for small fluctuations as

5 $${{\rm{\partial }}}_{t}\delta {\rm{\wp }}={a}_{0}({{\rm{\partial }}}_{x}^{2}-{{\rm{\partial }}}_{y}^{2})\delta {{\rm{\Pi }}}_{11}+2{a}_{0}{{\rm{\partial }}}_{x}{{\rm{\partial }}}_{y}\delta {{\rm{\Pi }}}_{12}+{D}_{{\rm{\wp }}}{{\rm{\nabla }}}^{2}\delta {\rm{\wp }},$$

6 $${{\rm{\partial }}}_{t}\delta {{\rm{\Pi }}}_{11}={\alpha }_{1}({{\rm{\wp }}}_{0})\delta {{\rm{\Pi }}}_{11}+{D}_{{\rm{\Pi }}}{{\rm{\nabla }}}^{2}\delta {{\rm{\Pi }}}_{11}+\frac{\beta }{2}({{\rm{\partial }}}_{x}^{2}-{{\rm{\partial }}}_{y}^{2})\delta {\rm{\wp }},$$

7 $${{\rm{\partial }}}_{t}\delta {{\rm{\Pi }}}_{12}={\alpha }_{1}({{\rm{\wp }}}_{0})\delta {{\rm{\Pi }}}_{12}+{D}_{{\rm{\Pi }}}{{\rm{\nabla }}}^{2}\delta {{\rm{\Pi }}}_{12}+\beta {{\rm{\partial }}}_{x}{{\rm{\partial }}}_{y}\delta {\rm{\wp }}.$$

Using Fourier transformation

8 $$Y({\rm{q}},\lambda )=\int {e}^{{\bf{iq}}{\bf{.r}}}{e}^{\lambda t}Y({\bf{r}},t)d{\bf{r}}dt$$

we get linear set of equations in the Fourier space as

9 $$\lambda (\begin{array}{l}\delta \wp \\ \delta {\Pi }_{11}\\ \delta {\Pi }_{12}\end{array})=M(\begin{array}{l}\delta \wp \\ \delta {\Pi }_{11}\\ \delta {\Pi }_{12}\end{array})$$

where *M* is the coefficient matrix as obtained from equations (5), (6), and (7) after the transformation. We solve equation (9) for the hydrodynamic modes *λ*. We choose ${q}_{x}={q}_{y}=\frac{q}{\sqrt{2}}$ since both the directions are equivalent. Therefore, we obtain

10 $$(\lambda -{\alpha }_{1}({{\rm{\wp }}}_{0})+{D}_{{\rm{\Pi }}}{q}^{2})\{(\lambda +{D}_{{\rm{\wp }}}{q}^{2})(\lambda -{\alpha }_{1}({{\rm{\wp }}}_{0})+{D}_{{\rm{\Pi }}}{q}^{2})-\frac{1}{2}{a}_{0}\beta {q}^{4}\}=0.$$

For small wave-vector *q*, we can find an unstable mode

11 $${\lambda }_{+}=-2{D}_{{\rm{\wp }}}{q}^{2}+\frac{{a}_{0}\beta {q}^{4}}{2|{\alpha }_{1}({{\rm{\wp }}}_{0})|}-\frac{{a}_{0}\beta {q}^{6}({D}_{{\rm{\Pi }}}-{D}_{{\rm{\wp }}})}{{\alpha }_{1}^{2}({{\rm{\wp }}}_{0})}.$$

For small ${D}_{\wp }$ and large actvitity *a*_0_, this mode becomes unstable for *q* < *q*_*c*_, where

12 $${q}_{c}^{2}=\frac{|{\alpha }_{1}({{\rm{\wp }}}_{0})|}{2{\rm{\Delta }}D}+\frac{1}{2}\sqrt{{(\frac{|{\alpha }_{1}({{\rm{\wp }}}_{0})|}{{\rm{\Delta }}D})}^{2}-\frac{8{D}_{{\rm{\wp }}}{\alpha }_{1}^{2}}{{\rm{\Delta }}D{a}_{0}\beta }},$$

provided ${\rm{\Delta }}D={D}_{{\rm{\Pi }}}-{D}_{\wp }$ is positive, and ${a}_{0}\beta \,\mathrm{ > \; 8}{D}_{\wp }{\rm{\Delta }}D$. Therefore, the unstable mode *λ*_+_ causes the I - BS transition for small diffusivity, i.e., at low temperature, and for large activity strength *a*_0_.

We also calculate the jump in the scalar order parameter *S* and the shift in the transition density from equations (3) and (4). A homogeneous steady state solution of these equations gives a mean field transition from the isotropic to the nematic state at density ${\wp }_{IN}$ where ${\alpha }_{1}(\wp )$ changes sign. Using renormalised mean field (RMF) method, we calculate an effective free energy ${ {\mathcal F} }_{eff}(S)$ close to the order-disorder transition where *S* is small. We consider density fluctuations $\delta \wp$ and neglect order parameter fluctuations. The effective free energy is

13 $${ {\mathcal F} }_{eff}(S)=-\frac{{b}_{2}}{2}{S}^{2}-\frac{{b}_{3}}{3}{S}^{3}+\frac{{b}_{4}}{4}{S}^{4}$$

where ${b}_{2}={\alpha }_{1}(\wp )+{\alpha }_{1}^{^{\prime} }c$, where *c* is a constant. ${\alpha }_{1}^{^{\prime} }=\partial {\alpha }_{1}/\partial \wp {|}_{{\wp }_{0}}$, ${b}_{3}=\frac{{a}_{0}{\wp }_{0}{\alpha }_{1}^{^{\prime} }}{2{D}_{\wp }}$, and ${b}_{4}=\frac{1}{2}{\wp }_{0}^{2}{\alpha }_{2}$. Both *b*_3_ and *b*_4_ are positive. A detailed calculation for ${ {\mathcal F} }_{eff}$ is shown in the Supplementary Information. The density fluctuations introduce a new cubic order term in the free energy ${ {\mathcal F} }_{eff}(S)$ that is proportional to the activity strength *a*_0_. The presence of such term produces a jump ${\rm{\Delta }}S={S}_{c}=\frac{2{b}_{3}}{3{b}_{4}}$ at a density ${\wp }_{c}={\wp }_{IN}(1-\frac{2{b}_{3}^{2}}{9{b}_{4}}) < {\wp }_{IN}$. Fluctuation in density produces a jump in order parameter and shifts the critical density. Such type of fluctuation induced transitions are called fluctuation dominated first order phase transitions in statistical mechanics^46^ and are widely studied for many systems^47, 48^. The jump in *S* and the shift in the transition density are proportional to the activity strength *a*_0_, and for *a*_0_ = 0 we recover the equilibrium transition.

## Discussion

In our present work we have introduced a minimal lattice model for the active nematic and study different ordering states in the density-temperature plane. A brief summary of the results is as follows. In the low density regime, the system is in the disordered isotropic (I) state with short range orientation correlation amongst the particles. In the low temperature regime, large density fluctuation in the active system induces a first order transition from the isotropic to the banded state with a jump in the scalar order parameter at a density lower than the equilibrium isotropic-nematic (I-N) transition density. The linear stability analysis of the isotropic state shows an instability for large activity strength in the low temperature regime. Such instability governs the band formation at density below the equilibrium I-N transition density. As we further increase density, bands vanish, and locally ordered patches appear in the inhomogeneous mixed (IM) state. Renormalised mean field calculation confirms the jump in the scalar order parameter and the shift in the transition density. With increasing temperature the shift in the transition density and the jump in scalar order parameter decrease, and no bands appear in the system. The IM state is a state with coexisting aligned and disordered domains, similar to the coexisting or defect-ordered states found in Refs 33, 34, 49–53.

In the high density regime, the active nematic shows switching between the IM (low *S*) and the homogeneous ordered (HO, high *S*) states, i.e., the system shows bistability. In the complete filling limit and with excluded volume assumption the active model reduces to the equilibrium model. Therefore, the active model tends to show a homogeneous nematic state in the high density regime. However, large activity strength makes the HO state unstable and leads the system to the IM state. This instability in the HO state is similar to the earlier studies in Refs 34 and 54. Ngo *et al*.^33^ considered a two dimensional off-lattice model for the active nematic without the exclusion constraint. In the low and moderate density regime, they show a homogeneous disordered phase and an inhomogeneous chaotic phase, which are similar to the isotropic and the IM states, respectively. Similar to their study, the spanning area of the IM state (golden regime in the phase diagram Fig. 1(a)) along the density axis decreases with the increasing temperature. In the high density limit, they note a homogeneous quasi-ordered phase only, which is similar to the HO state in our study. However, we show the bistability between the HO and the IM state in this density limit.

In conclusion, our lattice model for the active nematic is a simple one to design and execute numerically, and easy to compare with the corresponding equilibrium model. It shows new features like the BS in the low temperature regime and the bistability in the high density regime, as well as some of the early characterised states, e.g., the IM state. It also shows many basic features of the active nematic like large number fluctuation, long-time decay of orientation correlation, transition from SRO isotropic to QLRO nematic state. The shift in the transition density due to activity strength compared to the equilibrium model can be tested in experimental systems where activity can be tuned. We expect the emergence of the bistability in the high density regime in a two dimensional experimental system composed of apolar particles with finite dimension and high activity strength. It would be interesting to study the model without volume exclusion. In this study, particle orientation has continuous symmetry of *O*(2). Therefore, the equilibrium limit of our model is an apolar analogue of the two-dimensional XY-model. One can also study the model with discrete orientation symmetry as in Refs 20, 35, 36 and compare the results with the corresponding equilibrium model.

## Electronic supplementary material

Supplementary Information
