# Supplementary material for: Order-disorder transition in active nematic: A lattice model study
Source: Sci Rep. 2017 Aug 1;7:7080. doi: 10.1038/s41598-017-07301-w (PMC5539249; doi:10.1038/s41598-017-07301-w)
Supplement: Supplementary file 1 — Supplementary Information [file 41598_2017_7301_MOESM1_ESM.pdf]

Order-disorder transition in active nematic: A lattice model study

Rakesh Das,[1,*] Manoranjan Kumar,[1,†] and Shradha Mishra[1,2,‡]
[1] S N Bose National Centre for Basic Sciences, Block JD, Sector III, Salt Lake, Kolkata 700106, India
[2] Department of Physics, Indian Institute of Technology (BHU), Varanasi 221005, India

SUPPLEMENTARY FIGURE

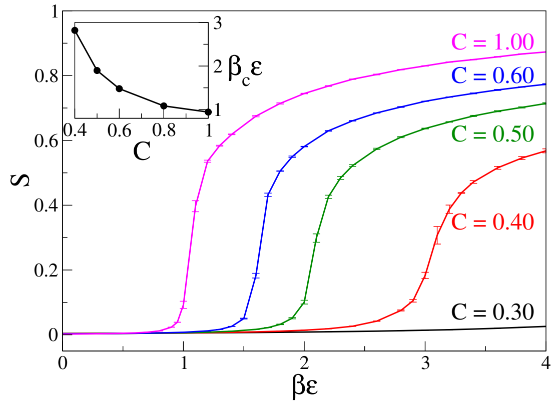

Figure S1. Order-disorder transition in the equilibrium model. Main - scalar order parameter S versus inverse temperature $\beta\epsilon$ plot for different density C. With increasing $\beta\epsilon$ the system goes from the isotropic (small S) to the nematic (large S) state. Inset - the critical inverse temperature decreases with increasing density.

SUPPLEMENTARY CALCULATION: RENORMALISED MEAN FIELD (RMF) STUDY OF ACTIVE NEMATIC FOR SMALL SCALAR ORDER PARAMETER

In this section we write an effective renormalised mean field free energy for the scalar order parameter S under the small S approximation. We consider the fluctuations in the density and ignore the order parameter fluctuations in the coupled hydrodynamic equations of motion for the active nematic. Density fluctuation introduces a cubic order term in S in the effective free energy. Such term produces a jump in S at a new transition density $\wp_c$ lower than the equilibrium I-N transition point $\wp_{IN}$. Shift in the transition density and the jump ΔS are directly proportional to the activity strength a_0. We recover the equilibrium limit for zero a_0.

In the main text we write the coupled hydrodynamic equations of motion for the density $\wp$ and the order parameter $\Pi = \wp\mathbf{Q}$ where nematic order parameter [1] is defined as

$$\mathbf{Q}(\mathbf{r}, t) = \frac{S}{2}\begin{pmatrix} \cos 2\theta(\mathbf{r}, t) & \sin 2\theta(\mathbf{r}, t) \\ \sin 2\theta(\mathbf{r}, t) & -\cos 2\theta(\mathbf{r}, t) \end{pmatrix} \tag{S1}$$

Here θ is the coarse-grained orientation at position $\mathbf{r}$ and time t. These hydrodynamic equations are previously derived in Ref. [2], but with specific coefficients. Here we retain general coefficients. The density equation is a continuity equation $\partial\wp/\partial t = -\nabla\cdot\mathbf{J}$, where the current $\mathbf{J}$ has two parts - active and diffusive. Details of these two currents are given in the main text. The activity strength a_0 represents the self-propelled nature of the particles, β is the coupling coefficient of the density in the order parameter equation, $D_\wp$ and D_Π are the diffusion coefficients in the density and the order parameter equations, respectively. $\alpha_1(\wp)$ and α_2 represent alignment in the system, and depend on the model parameters. For metric distance interacting model [2], $\alpha_1(\wp)$ is a function of density and changes sign

* rakesh.das@bose.res.in
† manoranjan.kumar@bose.res.in
‡ smishra.phy@itbhu.ac.in

at the critical density. We choose $\alpha_1(\wp) = \frac{\wp}{\wp_{IN}} - 1$ and $\alpha_2 = 1$. Let us consider a small perturbation $\delta\wp$ over the homogeneous steady state solution of the density equation so that $\wp = \wp_0 + \delta\wp$. Now from the density equation, we obtain

$$\begin{aligned} a_0\nabla_i\nabla_j\Pi_{ij} + D_\wp\nabla^2\delta\wp = 0 \\ \Rightarrow a_0\nabla_j\Pi_{ij} + D_\wp\nabla_i\delta\wp = \mathbf{c} \equiv constant \end{aligned} \tag{S2}$$

where $\Pi_{11} = -\Pi_{22} = \frac{S}{2}\cos(2\theta)$ and $\Pi_{12} = \Pi_{21} = \frac{S}{2}\sin(2\theta)$. Considering only the lowest order terms in S and θ, we obtain

$$\partial_x\delta\wp = -\frac{a_0\wp_0}{2D_\wp}\partial_x S \Rightarrow \delta\wp(x) = -\frac{a_0\wp_0}{2D_\wp}S + c_1 \tag{S3}$$

and

$$\partial_y\delta\wp = \frac{a_0\wp_0}{2D_\wp}\partial_y S \Rightarrow \delta\wp(y) = \frac{a_0\wp_0}{2D_\wp}S + c_2 \tag{S4}$$

Here we assume the system is aligned along one direction, and the variation in orientation is only along the perpendicular direction. Therefore, we can choose either of equations (S3) or (S4). Two constants c_1 and c_2 are the fluctuations in density when the nematic order parameter is zero.

Now from the equation for Π_{ij}, we obtain an effective equation for S as

$$\partial_t S = \left\{\alpha_1(\wp) - \frac{\wp^2}{2}\alpha_2 S^2\right\}S + \mathcal{O}(\nabla^2 S) + \mathcal{O}(\nabla^2\wp) \tag{S5}$$

We neglect all the derivative terms and retain only the polynomials in S, i.e., we neglect higher order fluctuations. The Taylor expansion of $\alpha_1(\wp)$ about the mean density $\wp_0$ gives $\alpha_1(\wp) = \alpha_1(\wp_0 + \delta\wp) = \alpha_1(\wp_0) + \alpha_1'\delta\wp$ where $\alpha_1' = \frac{\partial\alpha_1}{\partial\wp}|_{\wp_0}$. This gives

$$\partial_t S = \left\{\alpha_1(\wp_0) + \alpha_1'\delta\wp - \frac{\wp_0^2}{2}\alpha_2 S^2\right\}S \tag{S6}$$

We can write an effective free energy $\mathcal{F}_{eff}(S)$ so that

$$\partial_t S = -\frac{\delta\mathcal{F}_{eff}(S)}{\delta S} \tag{S7}$$

Substituting the expression for $\delta\wp$ from equation (S4), we obtain

$$-\frac{\delta\mathcal{F}_{eff}}{\delta S} = S\left\{\alpha_1(\wp_0) + \alpha_1'\left(\frac{a_0\wp_0}{2D_\wp}S + c_2\right) - \frac{\wp_0^2}{2}\alpha_2 S^2\right\} \tag{S8}$$

Therefore,

$$\mathcal{F}_{eff}(S) = -\frac{b_2}{2}S^2 - \frac{b_3}{3}S^3 + \frac{b_4}{4}S^4 \tag{S9}$$

where $b_2 = \alpha_1(\wp_0) + \alpha_1' c_2$, $b_3 = \frac{a_0\wp_0\alpha_1'}{2D_\wp}$ and $b_4 = \frac{1}{2}\wp_0^2\alpha_2$. Since the free energy is a state function, we have assumed the integration constant to be zero. Therefore, the fluctuation in the density introduces a cubic order term in the effective free energy $\mathcal{F}_{eff}(S)$. Effective free energy in equation (S9) is similar to the Landau free energy with a new cubic order term [3]. Now we calculate the jump ΔS and the new critical density from the coexistence condition for free energy. Steady state solutions of order parameter ($S = 0$ for isotropic and $S \neq 0$ for ordered state) are given by

$$\frac{\delta\mathcal{F}_{eff}}{\delta S} = \left(-b_2 - b_3 S + b_4 S^2\right)S = 0 \tag{S10}$$

Non-zero S is given by $-b_2 - b_3 S_c + b_4 S_c^2 = 0$. Coexistence condition implies

$$\mathcal{F}_{eff}(S_c) = \left(-\frac{b_2}{2} - \frac{b_3}{3}S_c + \frac{b_4}{4}S_c^2\right)S_c^2 = \mathcal{F}_{eff}(S = 0) = 0 \tag{S11}$$

Hence we get the solution

$$S_c = -\frac{3b_2}{b_3} \tag{S12}$$

and

$$b_2^c = -\frac{2b_3^2}{9b_4} \tag{S13}$$

Therefore, the jump at the new critical point is $\Delta S = \frac{2b_3}{3b_4}$. Since $b_4 > 0$ and hence $b_2^c < 0$, the new critical density

$$\wp_c = \wp_{IN}\left(1 - \frac{2b_3^2}{9b_4}\right) < \wp_{IN} \tag{S14}$$

is shifted to a lower density in comparison to the equilibrium transition density $\wp_{IN}$. Equation (S14) gives the expression for new transition density as given in the main text. Therefore, using renormalised mean field theory we find a jump ΔS at a lower density as compared to the equilibrium I-N transition density.

REFERENCES

[1] de Gennes, P. G. & Prost, J. *The Physics of Liquid Crystals* (Oxford: Clarendon Press, 1995).
[2] Bertin, E. *et al.* Mesoscopic theory for fluctuating active nematics. *New J. of Phys.* **15,** 085032 (2013).
[3] Chaikin, P. M. & Lubensky, T. C. *Principles of Condensed Matter Physics* (Cambridge: Cambridge University Press, 2000).
